# *Post-hoc* analysis of real-world retrospective study on neoadjuvant chemotherapy combined with immunotherapy for stage I-III non-small cell lung cancer

**DOI:** 10.3389/fimmu.2025.1576951

**Published:** 2026-01-02

**Authors:** Yanchi Shao, Shenghan Wang, Yingchun Yu, Weiwei Yang, Lei Song, Wuyuntanghesi, Yanbin Zhao

**Affiliations:** 1 Department of Medical Oncology, Harbin Medical University Cancer Hospital, Harbin, China; 2 Department of Cardiovascular Center, The First Hospital of Jilin University, Changchun, China; 3 Department of Immunology, Graduate School of Biomedical Sciences, Nagasaki University, Nagasaki, Japan; 4 Oujiang Laboratory (Zhejiang Lab for Regenerative Medicine, Vision and Brain Health), Wenzhou, China; 5 Cardio-Pulmonary Institute, Universities of Giessen and Marburg Lung Center (UGMLC), German Center for Lung Research (DZL), Justus-Liebig University Giessen, Giessen, Germany

**Keywords:** NSCLC, NLR, PLR, SII, PNI, prognosis

## Abstract

**Background and purpose:**

Real-world data on neoadjuvant therapy for stage I-III non-small cell lung cancer (NSCLC) is limited. This study evaluates the efficacy and safety of neoadjuvant chemotherapy alone versus chemotherapy combined with immunotherapy, focusing on pathological response, imaging outcomes, event-free survival (EFS), and adverse events. Additionally, a prognostic model for EFS is established.

**Methods:**

Data from 134 NSCLC patients who received neoadjuvant therapy were analyzed. Pathological response rates, objective response rate(ORR), EFS and adverse events were compared between the two groups. Independent prognostic factors were identified using logistic and Cox regression analyses, and a predictive model was constructed and evaluated using nomograms, Receiver Operating Characteristic(ROC) curves, calibration curves, and Decision Curve Analysis(DCA) curves.

**Results:**

1.The chemotherapy combined with immunotherapy group showed higher Pathological Complete Response (pCR) (48.8% vs. 12.5%) and ORR (80.2% vs. 47.9%) compared to the chemotherapy group (*P* < 0.05), with no difference in Major Pathological Response(MPR) or R0 resection rates. 2. Chemotherapy combined with immunotherapy group did not significantly increase TRAEs or ≥ Grade 3 TRAE rates, demonstrating acceptable safety. And incidence of immune-related adverse events (irAEs) was 20.9%, with ≥ Grade 3 irAEs at 4.3%. No treatment-related deaths occurred in neoadjuvant chemotherapy and chemotherapy combined with immunotherapy group. 3. Chemoimmunotherapy significantly prolonged EFS compared to chemotherapy alone (EFS not reached vs. 33 months, Hazard Ratio(HR)=0.45), reducing progression risk by 55% (*P* < 0.05).5.Pathological subtype was an independent predictor of pCR, with squamous cell carcinoma patients more likely to achieve pCR. 6. pCR and platelet-to-lymphocyte ratio (PLR) were independent prognostic factors for EFS in the chemoimmunotherapy group. The prognostic model showed good accuracy and clinical utility.

**Conclusion:**

Neoadjuvant chemotherapy combined with immunotherapy significantly improves pCR rates, ORR, and EFS compared to chemotherapy alone, with manageable adverse events. The predictive model provides valuable insights for clinical decision-making.

## 1 Introduction

According to 2025 data from the World Health Organization(WHO), lung cancer remains a leading cause of cancer incidence and mortality worldwide (1). NSCLC, accounting for approximately 85% of all lung cancer cases, is the predominant subtype (2). Neoadjuvant therapy, a preoperative treatment strategy, aims to downsize tumors, enhance resection rates, and improve patient prognosis (3). For patients with stage I-III NSCLC, surgery remains the primary treatment modality. However, due to postoperative recurrence and other factors, the five-year survival rate for these patients remains dismally low, underscoring the widespread adoption of neoadjuvant therapy in preoperative settings (4). Historically, neoadjuvant therapy approaches were relatively limited, with chemotherapy being the most commonly utilized strategy. In recent years, the advent of immune checkpoint inhibitors (ICIs) has expanded neoadjuvant options for stage I-III NSCLC. ICIs, either as monotherapy or in combination with chemotherapy, have become a cornerstone in the treatment of NSCLC (5).

Immune checkpoints, which are crucial in tumor immune evasion, refer to receptors and ligands expressed on the surface of immune cells and/or tumor cells. These molecules maintain immune homeostasis and prevent excessive immune activation. Tumor cells evade immune surveillance by expressing immunosuppressive ligands that bind to inhibitory receptors on T cells, such as Programmed Cell Death Protein1 (PD-1), Lymphocyte activation gene 3 (LAG-3), T cell immunoglobulin and mucin domain 3 (TIM-3),T cell immunoglobulin and ITIM domain (TIGIT), V-domain lg suppressor of T cell activation (VISTA) and CD244, forming co-inhibitory molecules that suppress T cell function (6). ICIs can block the formation of co-inhibitory molecules, thereby preserving T cell activity and enabling the effective recognition and elimination of tumor cells (6). Over the past few years, ICIs have rapidly developed and demonstrated promising results in various solid tumors. The most commonly studied immune checkpoints are PD-1/Programmed Cell Death Protein ligand 1 (PD-L1) and Cytotoxic T lymphocyte antigen 4 (CTLA-4). PD-1 is an immune inhibitory protein expressed on the surface of T cells. Anti-PD-1 drugs overactivate nonspecific T cells and simultaneously block both PD-L1 and Programmed cell death protein2 (PD-2). This suggests that anti-PD-1 therapies may exhibit greater efficacy even in tumors with low PD-L1 expression levels. PD-L1 is expressed on the surface of tumor cells, and anti-PD-L1 drugs act by blocking the regulatory pathways of tumor cell signaling. Consequently, anti-PD-L1 drugs are theoretically associated with a lower incidence or milder severity of irAEs (7). CTLA-4 is expressed on activated CD4+ and CD8+ T cells, with CTLA-4-targeting antibodies including ipilimumab and tremelimumab (8).

Immunotherapy has been widely applied in patients with advanced NSCLC, significantly extending survival and improving quality of life. However, the real-world efficacy and safety of immunotherapy as part of neoadjuvant treatment for patients with potentially resectable stage I–III NSCLC remain to be fully validated. Moreover, the benefits of immunotherapy in this patient subgroup are not universal. Identifying cost-effective and convenient prognostic factors for predicting patient outcomes is crucial. Existing studies suggest that circulating tumor DNA (ctDNA) (7), PD-L1 expression (9), tumor mutational burden (TMB) (10), and the transcriptomic characteristics of Fc Receptor-Like 4 Positive (FcRL4+) Fc Receptor-Like 5 Positive (FcRL5+) memory B cells and CD16+ CX3CR1+ monocytes (11) may serve as predictors of response to immunotherapy. However, the clinical application of these biomarkers is limited by their high costs and complex acquisition processes. Recent research indicates that hematological and nutritional serum indicators could potentially predict the efficacy of immunotherapy (12). In this study, we compared the efficacy of neoadjuvant chemotherapy alone versus neoadjuvant chemotherapy combined with immunotherapy. Additionally, we closely monitored adverse events in the combination therapy group during treatment. Using statistical methods, we conducted univariate and multivariate. Analyses to assess the effects of clinical characteristics and markers such as NLR, PLR,SII,PNI, and COUNT score on patient prognosis. Finally, we constructed a nomogram based on multivariate Cox regression results and evaluated its predictive performance.

## 2 Materials and methods

### 2.1 Object of study

We retrospectively collected data from 134 patients with NSCLC who underwent lung cancer resection at Harbin Medical University Cancer Hospital after receiving neoadjuvant therapy between May 2018 and December 2023. Basic clinical information, examination findings, and laboratory test results were gathered for each patient.

#### 2.1.1 Inclusion criteria

(1) Pathologically confirmed diagnosis of NSCLC. (2) Tumor staging: Stage I–III. (3) Age ≥ 18 years. (4) Eastern Cooperative Oncology Group (ECOG) performance status score: 0–1. (5) Patients who received at least one cycle of neoadjuvant immunotherapy combined with chemotherapy or neoadjuvant chemotherapy alone before undergoing lung cancer resection.

#### 2.1.2 Exclusion criteria

(1) Patients with organ dysfunction who were unable to tolerate lung resection surgery or those who opted to forgo surgical treatment for personal reasons.(2) Patients with a history of prior antitumor treatment.(3) Patients with incomplete case information.(4)Genetic testing showed Epidermal growth factor receptor (EGFR) and Anaplastic lymphoma kinase (ALK) mutations in the patient.

### 2.2 Collection of clinical data

#### 2.2.1 Basic information

Patient demographic and clinical data, including sex, age, BMI, smoking and alcohol consumption history, pathological type, tumor location, tumor stage, R0 resection status, number of neoadjuvant therapy cycles, initial treatment date, and time to first disease progression, were collected using the clinical data management system.

#### 2.2.2 Peripheral blood data

Peripheral blood data, including complete blood count and liver and kidney function tests within one week before the initiation of neoadjuvant therapy, were recorded. Specific indices were calculated using the following formulas: NLR: Neutrophil count/Lymphocyte count. PLR: Platelet count/Lymphocyte count. SII: Neutrophil count (10^9^/L)×Platelet count (10^9^/L)/Lymphocyte count (10^9^/L).PNI: Serum albumin (g/L)+5×Total peripheral lymphocyte count (10^9^/L).COUNT score: Calculated based on lymphocyte count, serum albumin level, and total cholesterol level as follows:①Lymphocyte count: >1.6×10^9^/L=0points; (1.2–1.6) ×10^9^/L=1point; (0.8–1.1) ×10^9^/L = 2 points; <0.8×10^9^/L=3 points.②Serum albumin level: ≥35 g/L= 0 points; 30–34 g/L=2 points; 25–29g/L= 4 points; <25 g/L = 6 points.③Total cholesterol level: ≥4.65 mmol/L=0 points; 3.62–4.64 mmol/L=1 point; 2.58–3.61 mmol/L=2 points; <2.58 mmol/L=3 points. The total COUNT score (range: 0–12) was calculated by summing the scores from the above three indicators. Higher scores indicate poorer nutritional status:9–12: Severe malnutrition.5–8: Moderate malnutrition.2–4: Mild malnutrition.0–1: Normal nutritional status. Patients were further categorized into low COUNT score group (0–1 points) and high COUNT score group (≥ 2 points).

### 2.3 Efficacy assessment

#### 2.3.1 Pathological response evaluation criteria

MPR: Defined as ≤10% residual viable tumor cells in tumor tissue on pathological slides following neoadjuvant therapy. pCR: Defined as no residual viable tumor cells detected on pathological slides after neoadjuvant therapy. According to the 2022 guidelines of the Chinese Society of Clinical Oncology (CSCO), R0 resection is defined by the following criteria: (1) Negative surgical margins, including bronchus, artery, vein, peribronchial, and tissues adjacent to the tumor. (2) Dissection of at least six lymph node stations, with three from the intrapulmonary region and three from the mediastinum, mandatorily including station (3) Microscopic examination of the highest resected lymph node confirms no residual tumor. (4) No extracapsular extension of lymph node metastasis.

#### 2.3.2 Clinical response evaluation criteria

Chest CT scans were performed before and after neoadjuvant therapy to assess imaging results according to the Response Evaluation Criteria in Solid Tumours (RECIST) criteria. Tumor changes were evaluated by calculating the ratio of the difference between the maximum diameter of the primary lesion before and after treatment to the pre-treatment tumor diameter. Treatment responses were categorized as follows: Complete Response (CR): Disappearance of all lesions. Partial Response (PR): A ≥30% decrease in the sum of the diameters of all target lesions. Progressive Disease (PD): A ≥20% increase in the sum of the diameters of all target lesions or the appearance of new lesions. Stable Disease (SD): Tumor changes that do not meet the criteria for PR or PD, i.e., a reduction of <30% or an increase of <20%.The ORR was calculated as the sum of CR% and PR%.

#### 2.3.3 Adverse events

TRAEs: These refer to all adverse reactions occurring during drug treatment that are caused by the treatment itself, rather than by other diseases or factors in the patient. irAE: Adverse events caused by immunotherapy, usually resulting from excessive activation of the immune system or autoimmune reactions triggered by immune checkpoint inhibition. TRAEs are evaluated based on the National Cancer Institute Common Terminology Criteria for Adverse Events (NCI-CTCAE) version 5.0 and are categorized into grades 1 to 5.

#### 2.3.4 Follow-up

Patients included in the study were followed up through electronic medical records, appointment records, telephone interviews, return visits, and subsequent consultations. The follow-up cutoff date was September 30, 2024.

#### 2.3.5 Study endpoints

The primary endpoint of this study was the pathological response rate (MPR or pCR). The secondary endpoint was EFS, defined as the time from the initiation of neoadjuvant therapy to disease progression or death from any cause.

#### 2.3.6 Statistical methods

Data were processed using SPSS version 26.0 and R version 4.2.3. The specific statistical methods are as follows: 1.Descriptive Statistics:(1)Continuous variables with a normal distribution are expressed as the mean ± standard deviation (X ± s).(2)The t-test was used for comparisons between chemotherapy group and the chemotherapy combined with immunotherapy group.(3)Categorical variables are presented as rates (%) and compared between groups using the χ² test or Fisher’s exact test, as appropriate.(4)For non-normally distributed continuous variables and ordinal data, the rank-sum test was applied. 2.Logistic Regression Analysis: Variables with *P* < 0.05 from univariate analyses were included in multivariate analyses. Odds ratios (OR) and 95% confidence intervals (CI) were calculated. 3.ROC Curve Analysis: ROC curves were constructed by plotting sensitivity on the y-axis and (1 - specificity) on the x-axis to evaluate the effectiveness of the target variable. The area under the ROC curve (AUC) was used as a measure of predictive value: AUC = 1: Excellent predictive value.0.7 ≤ AUC < 1: High predictive value, superior to random chance.0.5 ≤ AUC < 0.7: Low predictive value. AUC ≤ 0.5: No predictive capability. The optimal cutoff value was determined using the maximum Youden Index.4.Kaplan-Meier Analysis and Cox Regression: Kaplan-Meier survival curves were used to evaluate EFS differences among patients, with the log-rank test used for group comparisons. Cox regression analysis was employed to identify independent prognostic factors associated with EFS.5.Nomogram and Calibration Curve: Independent prognostic factors identified through multivariate Cox regression were included in a nomogram, constructed using the “nomogram” function in R. The “calibrate” function was used to draw calibration curves by comparing predicted risk values with observed outcomes. The closer the calibration curve is to the diagonal, the better the model’s predictive accuracy. The concordance index (C-index) was used to evaluate the discrimination ability of the clinical prediction model, with values ranging from 0.5 to 1. A C-index value closer to 1 indicates better predictive performance of the model. Time-Dependent ROC and Decision Curve Analysis: The “timeROC” function was used to generate time-dependent ROC curves. When a variable (risk factor) shows a trend toward promoting the occurrence of events, its AUC > 0.5. A larger AUC (closer to 1) indicates better prognostic efficacy and higher accuracy. The “dca” function was used to draw DCA curves, which evaluate the model’s clinical utility. If the model’s curve consistently remains above the “all positive” and “all negative” lines within a specific range of x-values, it indicates practical applicability of the model. All statistical tests were two-tailed, and a *P*-value < 0.05 was considered statistically significant.

## 3 Results

### 3.1 Baseline characteristics of the neoadjuvant chemotherapy combined with immunotherapy group and the neoadjuvant chemotherapy group

A total of 134 NSCLC patients who underwent preoperative neoadjuvant therapy were included in this study. Among them, 48 patients received neoadjuvant chemotherapy alone, and 86 patients received neoadjuvant chemotherapy combined with immunotherapy. Statistical analysis revealed no significant differences in baseline clinical characteristics, smoking and alcohol consumption history, or tumor staging between the chemotherapy group and the chemotherapy combined with immunotherapy group (*P* > 0.05) (**Table 1**).

**Table 1:** Baseline clinical characteristics of the chemotherapy group and the chemotherapy combined with immunotherapy group.

Clinical data		Chemotherapy group (n=48)	Chemotherapy+immunotherapy (n=86)	*P*
Age (n,%)	≥60	26 (54.2%)	46 (53.5%)	0.892
	<60	22 (45.8%)	40 (46.5%)	
Gender (n,%)	Male	35 (72.9%)	64 (74.4%)	0.849
	Female	13 (27.1%)	22 (25.6%)	
BMI (n,%)	≥24	19 (39.6%)	43 (50%)	0.246
	<24	29 (60.4%)	43 (50%)	
Pathological type (n,%)	Squamous cell carcinoma	37 (77.1%)	63 (73.3%)	0.625

	Adenocarcinoma	11 (22.9%)	23 (26.7%)	
Tumor stage (n,%)	Stage I	2 (4.2%)	7 (8.2%)	0.182
	Stage II	7 (14.6%)	22 (25.6%)	
	Stage III	39 (81.3%)	57 (66.2%)	
Treatment cycle (n,%)	≥3	42 (87.5%)	68 (79.1%)	0.222
	<3	6 (12.5%)	18 (20.9%)	
R0 resection (n,%)	Yes	43 (89.6%)	80 (93%)	0.196
	No	5 (10.4%)	6 (7%)	
ECOG (n,%)	0	9 (18.8%)	17 (19.8%)	0.866
	1	39 (81.3%)	69 (80.2%)	
Alcohol history (n,%)	Yes	12 (25.0%)	21 (24.4%)	0.940
	No	36 (75.0%)	65 (75.6%)	
Smoking history (n,%)	Yes	23 (47.9%)	39 (45.3%)	0.775
	No	25 (52.1%)	47 (54.7%)	

### 3.2 Comparison of pathological efficacy between neoadjuvant chemotherapy combined with immunotherapy and neoadjuvant chemotherapy

Among the 86 patients who underwent neoadjuvant chemotherapy combined with immunotherapy, 42 patients (48.8%) achieved pCR, while 44 patients (51.2%) did not. In terms of MPR, 31 patients (36%) were classified as MPR, while 55 patients (64%) were non-MPR. Among the 48 patients who underwent neoadjuvant chemotherapy alone, 6 patients (12.5%) achieved pCR, while 42 patients (87.5%) did not. In terms of MPR, 13 patients (27.1%) were classified as MPR, while 35 patients (72.9%) were non-MPR. Statistical analysis using the chi-square test showed no significant difference in MPR between the two groups (*P* = 0.386,*P* > 0.05); however, a significant difference in pCR was observed (*P* = 0.000,*P* < 0.05).

### 3.3 Comparison of clinical efficacy between neoadjuvant chemotherapy combined with immunotherapy and neoadjuvant chemotherapy

According to RECIST criteria, 69 patients (80.2%) in the chemotherapy combined with immunotherapy group achieved PR, and 17 patients (19.8%) showed SD, resulting in an ORR of 80.2%. In the chemotherapy-only group, 23 patients (47.9%) achieved PR, 24 patients (50%) showed SD, and 1 patient (2.1%) experienced PD, resulting in an ORR of 47.9%. The difference in ORR between the two groups was statistically significant (*P* = 0.000,*P* < 0.05). Additionally, 80 patients (93%) in the chemotherapy combined with immunotherapy group underwent R0 resection, while 43 patients (89.6%) in the chemotherapy-only group achieved R0 resection. There was no statistically significant difference in R0 resection rates between the two groups (*P* = 0.713*,P* > 0.05).

### 3.4 Adverse events during treatment in the neoadjuvant chemotherapy and chemotherapy combined with immunotherapy groups

The incidence of TRAEs in the Chemotherapy Combined with Immunotherapy group was 50% (43 cases), slightly higher than the 39.6% (19 cases) in the chemotherapy-only group, but the difference between the two groups was not statistically significant (*P* = 0.328, *P*>0.05). The incidence of ≥Grade 3 TRAE was similar in both groups, 17.4% (15 cases) and 16.7% (8 cases), respectively, with no statistically significant difference (*P* = 1.000, *P*>0.05). No treatment-related deaths occurred in either group, but 2 patients (2.3%) in the Chemotherapy Combined with Immunotherapy group discontinued treatment due to TRAEs, while no such cases occurred in the chemotherapy group, with no significant difference (*P* = 0.748, *P*>0.05). Additionally, the overall incidence of irAE in the Chemotherapy Combined with Immunotherapy group was 20.9% (18 cases), with a ≥Grade 3 irAE incidence of 4.3% (5 cases). These included 1 case of immune-related pneumonia, 3 cases of hypothyroidism, and 1 case of hypophysitis. All patients recovered to Grade 1 after discontinuation of immunotherapy and steroid treatment. This suggests that neoadjuvant Chemotherapy Combined with Immunotherapy has a favorable safety profile. No deaths or treatment discontinuations due to irAEs occurred in either group (**Table 2**).

**Table 2:** Adverse events during treatment in the neoadjuvant chemotherapy and chemotherapy combined with immunotherapy groups.

Adverse reaction	Chemotherapy group (N=48)	Chemotherapy + immunotherapy group (N=86)
Any level of TRAE	19 (39.6%)	43 (50%)
≥ Grade 3 TRAE	8 (16.7%)	15 (17.4%)
TRAE leading to death	0 (0%)	0 (0%)
TRAE leading to treatment discontinuation	0 (0%)	2 (2.3%)
Any level of irAE	0 (0%)	18 (20.9%)
≥ Grade 3 irAE	0 (0%)	5 (4.3%)
irAE leading to death	0 (0%)	0 (0%)
irAE leading to treatment discontinuation	0 (0%)	0 (0%)

### 3.5 Comparison of survival between neoadjuvant immunochemotherapy and neoadjuvant chemotherapy

**Figure 1:**
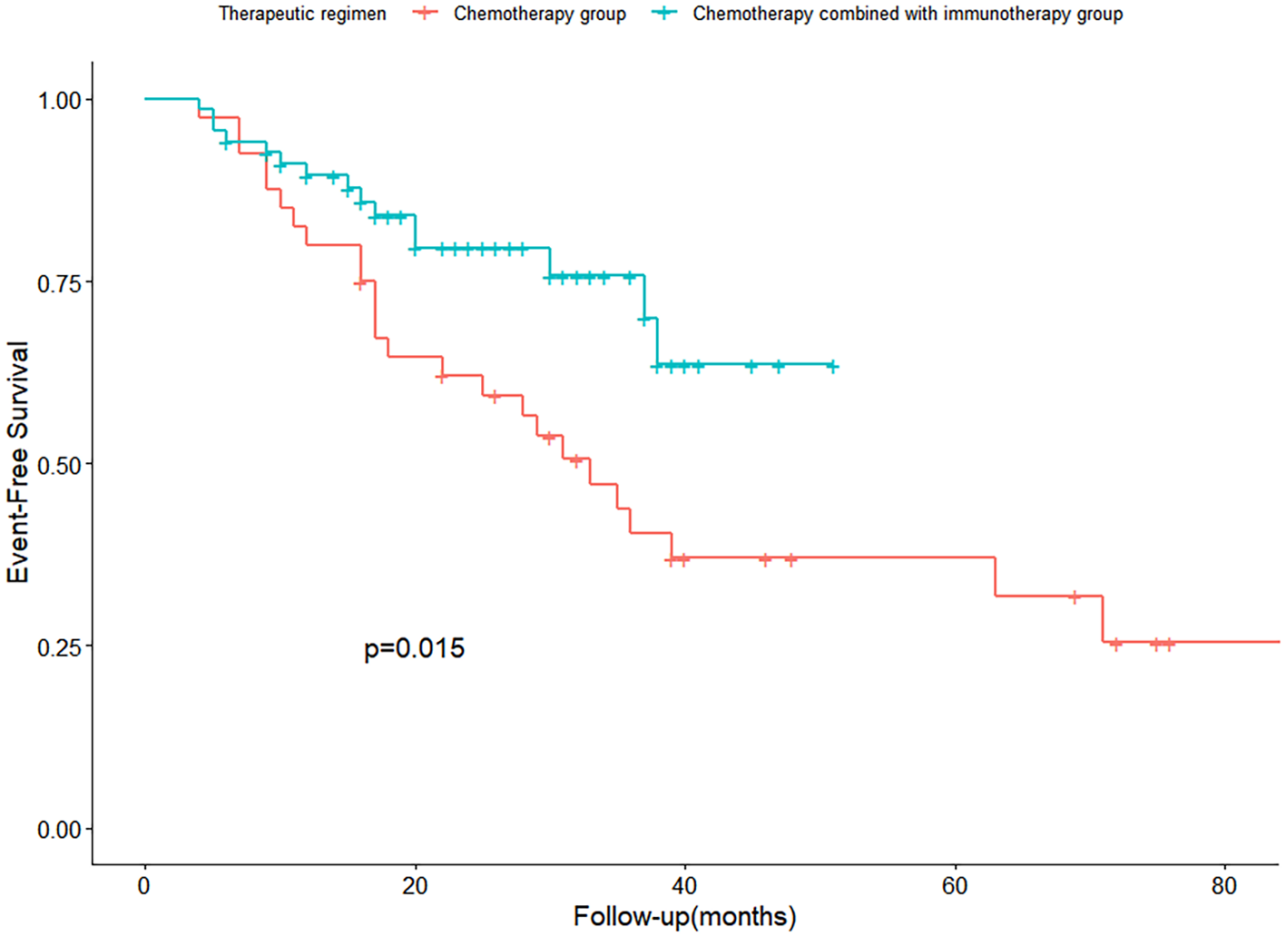
K-M survival curves comparing neoadjuvant chemotherapy and chemotherapy combined with immunotherapy groups.

As of the last follow-up on September 30, 2024, the K-M survival curves of EFS for the chemotherapy group and the chemotherapy combined with immunotherapy group were plotted, and a log-rank test was performed. The results showed a significant difference between the two groups at different levels (*P* = 0.015, *P* < 0.05).In the chemotherapy combined with immunotherapy group, among 86 patients, 18 were lost to follow-up, 15 experienced disease progression, and 53 had no disease progression as of the follow-up deadline. The median EFS of this group has not yet been reached. In the neoadjuvant chemotherapy group, among 48 patients, 8 were lost to follow-up, and of the remaining 40 patients, 25 experienced disease progression, while 15 did not show disease progression before the follow-up deadline. The neoadjuvant chemotherapy combined with immunotherapy group significantly prolonged the median EFS (EFS not reached vs. control group 33 months, HR = 0.45(95%CI:0.24-0.87)), reducing the risk of disease progression, recurrence, or metastasis by 55% (**Figure 1**).

### 3.6 Subgroup analysis of the neoadjuvant chemotherapy combined with immunotherapy group

#### 3.6.1 Clinical features of pCR vs non-pCR in the chemotherapy combined with immunotherapy group

The clinical characteristics of the pCR and non-pCR subgroups in the chemotherapy combined with immunotherapy group were compared. Statistical analysis revealed significant differences in gender, pathological type between the two groups (P<0.05). However, there were no significant differences in age, ECOG score, smoking history, etc. (P>0.05) (**Table 3**).

**Table 3:** Clinical features of pCR and non-pCR groups.

Clinical data		pCR group	Non-pCR group	*P*
Gender (n,%)	male	37 (88.1%)	27 (64.1%)	0.005
	female	5 (11.9%)	17 (38.6%)	
Age (n,%)	≥60	22 (52.4%)	23 (52.3%)	0.992
	<60	20 (47.6%)	21 (47.7%)	
ECOG (n,%)	0	10 (23.8%)	7 (15.9%)	0.358
	1	32 (76.2%)	37 (84.1%)	
Smoking history (n,%)	No	19 (45.2%)	28 (63.6%)	0.087
	Yes	23 (54.8%)	16 (36.4%)	
Alcohol history (n,%)	No	30 (71. 4%)	35 (79.5%	0.381
	Yes	12 (28.6%)	9 (20.5%)	
BMI (n,%)	<24	21 (50.0%)	22 (50%)	1.000
	≥24	21 (50.0%)	22 (50%)	
Pathological type (n,%)	Squamous cell carcinoma	36 (85.7%)	27 (61.4%)	0.000
	Adenocarcinoma	6 (14.3%)	17 (38.6%)	
Tumor location (n,%)	Left	21 (32.3%)	16 (36.4%)	0.202
	Right	21 (67.7%)	28 (50.9%)	
Therapeutic evaluation (n,%)	SD	2 (4.8%)	15 (34.1%)	0.001
	PR	40 (95.2%)	29 (65.9%)	
R0 resection (n,%)	Yes	41 (97.6%)	39 (88.6%)	0.226
	No	1 (2.4%)	5 (11.4%)	
Tumor stage (n,%)	Stage I	5 (11.9%)	2 (4.5%)	0.362
	Stage II	9 (21.5%)	13 (29.6%)	
	Stage III	28 (66.7%)	29 (65.9%)	
T (n,%)	1	3 (7.1%)	3 (6.8%)	0.733
	2	25 (59.5%)	22 (50.0%)	
	3	10 (23.8%)	15 (34.1%)	
	4	4 (9.5%)	4 (9.1%)	
N (n,%)	0	8 (19.0%)	8 (18.2%)	
	1	8 (19.0%)	13 (29.5%)	0.707
	2	22 (52.4%)	20 (45.5%)	
	3	4 (9.5%)	3 (6.8%)	

#### 3.6.2 Clinical features of MPR vs non-MPR in the chemotherapy combined with immunotherapy group

The clinical features of the MPR and non-MPR subgroups in the chemotherapy combined with immunotherapy group were compared. Statistical analysis showed no significant differences between the two groups in terms of smoking, alcohol history, or tumor stage (*P*>0.05) (**Table 4**).

**Table 4:** Clinical features of MPR and non-MPR groups.

Clinical data		MPR group	Non-MPR group	*P*
Gender (n,%)	Male	21 (67.7%)	43 (78.2%)	0.287
	Female	10 (32.2%)	12 (21.8%)	
Age (n,%)	≥60	15 (48.4%)	30 (54.5%)	0.583
	<60	16 (51.6%)	24 (45.5%)	
ECOG (n,%)	0	5 (16.1%)	12 (21.8%)	0.525
	1	26 (83.9%)	43 (78.2%)	
Smoking history (n,%)	No	18 (58.1%)	29 (52.7%)	0.766
	Yes	13 (41.9%)	26 (47.3%)	
Alcohol history (n,%)	No	24 (77.4%)	41 (74.5%)	0.822
	Yes	7 (22.6%)	14 (25.5%)	
BMI (n,%)	<24	16 (51.6%)	27 (49.1%)	0.883
	≥24	15 (48.4%)	28 (50.9%)	
Pathological type (n,%)	Squamous cell carcinoma	23 (74.2%)	40 (72.7%)	0.883
	Adenocarcinoma	8 (25.8%)	15 (27.3%)	
Tumor location (n,%)	Left	10 (32.3%)	27 (49.1%)	0.13
	Right	21 (67.7%)	28 (50.9%)	
Therapeutic evaluation (n,%)	SD	8 (25.8%)	9 (16.4%)	0.993
	PR	23 (74.2%)	46 (83.6%)	
R0 resection (n,%)	Yes	29 (93.5%)	51 (92.7%)	0.886
	No	2 (6.5%)	4 (7.3%)	
Tumor stage (n,%)	Stage I	2 (6.5%)	5 (9.1%)	0.555
	Stage II	10 (32.2%)	12 (21.8%)	
	Stage III	19 (61.3%)	38 (69.1%)	
T (n,%)	1	1 (3.2%)	5 (9.1%)	0.210
	2	14 (45.2%)	33 (60%)	
	3	12 (38.7%)	13 (23.6%)	
	4	4 (12.9%)	4 (7.3%)	
N (n,%)	0	8 (25.8%)	8 (14.5%)	0.206
	1	10 (32.3%)	11 (20%)	
	2	11 (35.5%)	31 (56.4%)	
	3	2 (6.5%)	5 (9.1%)	

#### 3.6.3 Univariate analysis of factors associated with pathological response in the neoadjuvant chemotherapy combined with immunotherapy group

Univariate analysis was performed using clinical features such as gender, age, tumor location, stage, NLR, PLR, SII, PNI (with the optimal cutoff value determined by ROC curve), and COUNT score. The results showed that pathological type and gender were independent prognostic factors for pCR (*P* < 0.05) (**Table 5**). Furthermore, univariate analysis of the same factors did not identify any significant independent prognostic factors for MPR.

**Table 5:** Univariate analysis of factors associated with pCR in neoadjuvant chemotherapy combined with immunotherapy.

Variable	pCR	Non-pCR	*P*
Age (n,%)			0.992
≥60	22 (52.4%)	23 (52.3%)	
<60	20 (47.6%)	21 (47.7%)	
ECOG (n,%)			0.36
0	10 (23.8%)	7 (15.9%)	
1	32 (76.2%)	37 (84.1%)	
Smoking history (n,%)			0.089
No	19 (45.2%)	28 (63.6%)	
Yes	23 (54.8%)	16 (36.4%)	
Alcohol history (n,%)			0.383
No	30 (71.4%)	35 (79.5%)	
Yes	12 (28.6%)	9 (20.5%)	
BMI (n,%)			1.000
<24	21 (50%)	22 (50%)	
≥24	21 (50%)	22 (50%)	
Tumor location (n,%)			0.203
Left	21 (50%)	16 (36.4%)	
Right	21 (50%)	28 (63.6%)	
R0 resection (n,%)			0.138
Yes	41 (97.6%)	39 (88.6%)	
No	1 (2.4%)	5 (11.4%)	
Pathological type (n, %)			0.014
Squamous cell carcinoma	36 (85.7%)	27 (61.4%)	
Adenocarcinoma	6 (14.3%)	17 (38.6%)	
Gender (n, %)			0.007
Male	37 (88.1%)	27 (64.1%)	
Female	5 (11.9%)	17 (38.6%)	
Tumor stage (n, %)			0.315
Stage I	5 (11.9%)	2 (4.5%)	
Stage II	9 (21.5%)	13 (29.6%)	
Stage III	28 (66.7%)	29 (65.9%)	
NLR (n, %)			0.137
≥2.53	21 (50%))	15 (34.1%)	
<2.53	21 (50%)	29 (65.9%)	
PLR (n, %)			0.973
≥67.09	41 (97.6%)	43 (97.7%)	
<67.09	1 (2.4%)	1 (2.3%)	
SII (n, %)			0.172
≥913.27	13 (31%)	8 (18.2%)	
<913.27	29 (69%)	36 (81.8%)	
PNI (n, %)			0.194
≥48.38	33 (78.6%)	29 (65.9%)	
<48.38	9 (21.4%)	15 (34.1%)	
COUNT score			0.912
Low score group	33 (78.6%)	35 (79.5%)	
High score group	9 (21.4%)	9 (20.5%)	

#### 3.6.4 Multivariate analysis of factors associated with pCR in the neoadjuvant chemotherapy combined with immunotherapy group

Following statistical analysis, the factors that showed significant differences in univariate analysis for pCR were included in the multivariate analysis. The results indicated that pathological type was an independent prognostic factor for pCR (*P* < 0.05) (**Table 6**). Since no statistically significant factors were identified in the univariate analysis for MPR, no multivariate analysis was performed for MPR.

**Table 6:** Multivariate analysis of factors associated with pCR in neoadjuvant chemotherapy combined with immunotherapy.

Variable	B coefficient	Standard error	WaldX2 value	OR value	95%CI		*P*
					Lower	Upper	
Pathological type	1.333	0.54	6.102	3.793	1.317	10.925	0.014
Gender	-0.65	0.45	0.021	0.937	0.388	2.262	0.885

#### 3.6.5 Predictive value of NLR, PLR, SII, and PNI for pCR and MPR in the neoadjuvant chemotherapy combined with immunotherapy group

**Figure 2:**
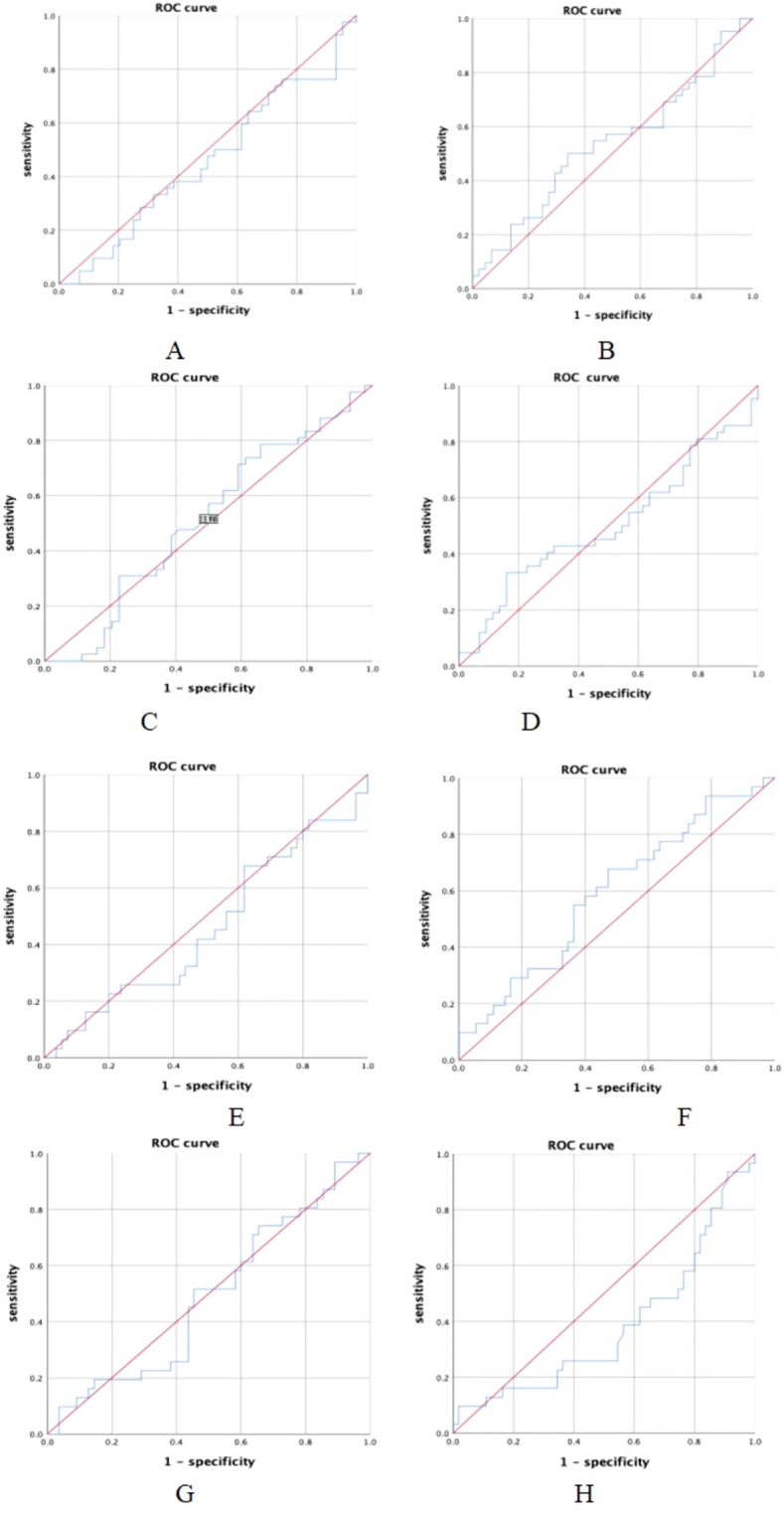
ROC curves for NLR, PLR, SII, PNI. **(A)** NLR and pCR(AUC=0.535,*P* = 0.58); **(B)** PLR and pCR(AUC=0.457,*P* = 0.489); **(C)** SII and pCR(AUC=0.504,*P* = 0.945); **(D)** PNI and pCR(AUC=0.504,*P* = 0.945); **(E)** NLR and MPR(AUC=0.462,*P* = 0.556; **(F)** PLR and MPR(AUC=0.606 *P* = 0.103); **(G)** SII and MPR(AUC=0.493,*P* = 0.918); **(H)** PNI and MPR(AUC=0.395,*P* = 0.107).

ROC curves were plotted for NLR, PLR, SII, and PNI in relation to pCR and MPR (**Figure 2**). The areas under the ROC curves for both MPR and pCR were relatively small, indicating that the pre-treatment NLR, PLR, SII, and PNI had limited predictive value for MPR and pCR in NSCLC patients receiving neoadjuvant immunochemotherapy.

#### 3.6.6 Predictive value of PLR, NLR, SII, and PNI for EFS in the neoadjuvant chemotherapy combined with immunotherapy group

**Figure 3:**
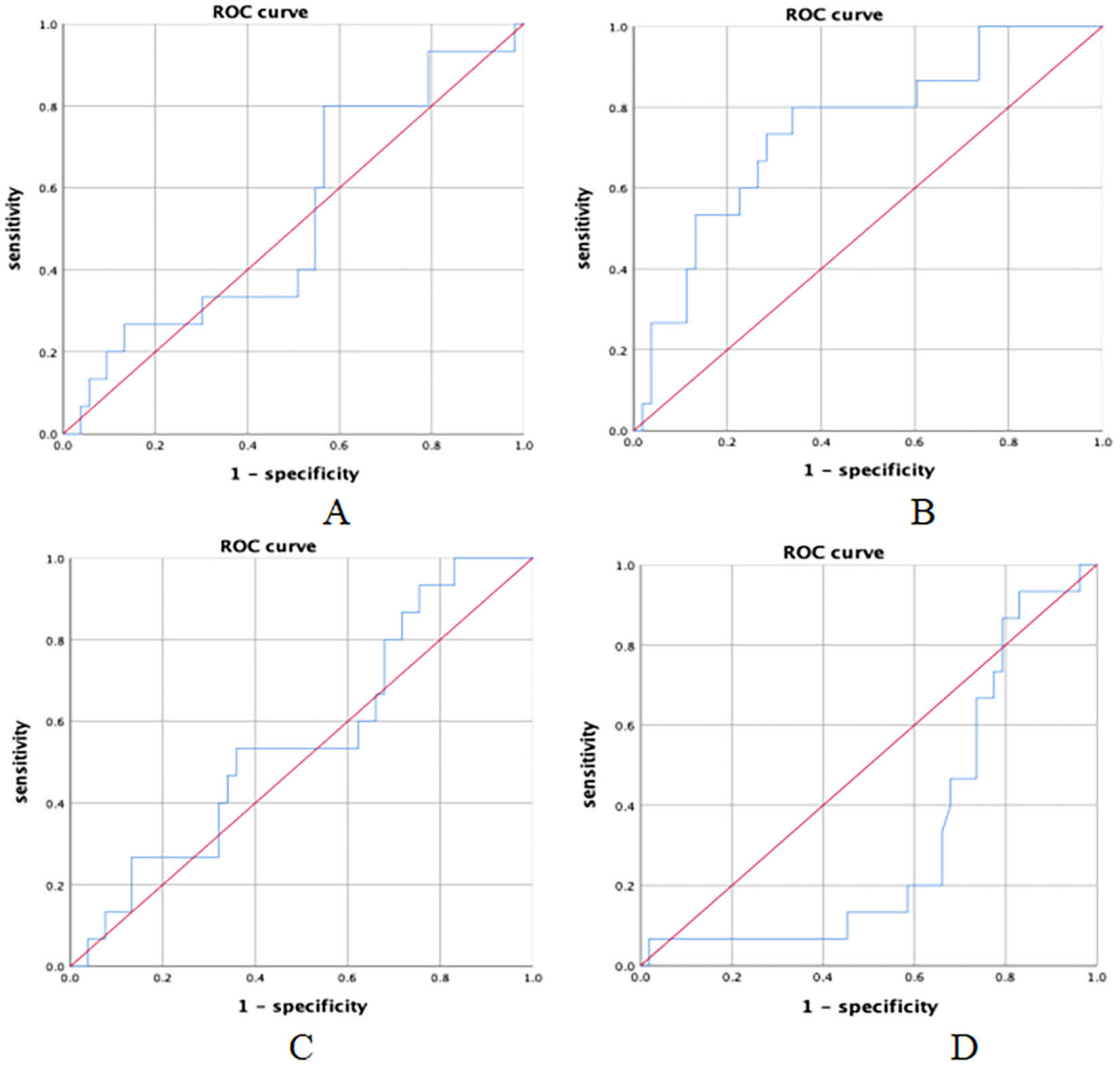
ROC curves for NLR, PLR, SII, PNI. **(A)** NLR and EFS(AUC=0.531,*P* = 0.717); **(B)** PLR and EFS(AUC=0.746,*P* = 0.004); **(C)** SII and EFS(AUC=0.556,*P* = 0.510); **(D)** PNI and EFS(AUC=0.328,*P* = 0.043).

ROC curve analysis was used to assess the predictive value of PLR, NLR, SII, and PNI for EFS (**Figure 3**). The results indicated that pre-treatment NLR, SII, and PNI had limited predictive value for EFS in NSCLC patients receiving neoadjuvant immunochemotherapy. However, PLR demonstrated a relatively better predictive value for EFS.

#### 3.6.7 Cox regression analysis of risk factors affecting EFS in the neoadjuvant chemotherapy combined with immunotherapy group

Clinical data, including age, gender, BMI, smoking and alcohol history, tumor stage, pathological type, NLR (with 2.09 as the cutoff value for grouping), PLR (with 139.72 as the cutoff value for grouping), SII (with 359.16 as the cutoff value for grouping), PNI (with 45.4 as the cutoff value for grouping), and COUNT score, were included in Cox regression analysis. Univariate analysis showed that factors such as R0 resection, MPR, pCR, and PLR were independent prognostic factors for EFS (*P* < 0.05). These factors were further included in the multivariate Cox regression analysis, which indicated that pCR and PLR were independent prognostic factors for EFS (*P* < 0.05) (**Tables 7**, **8**).

**Table 7:** Univariate analysis of EFS.

Clinical features		Univariate Analysis		
		HR	95%CI	*P*
Gender	(male, female)	0.64	0.218-1.879	0.417
Age	(60 or ≥60)	0.685	0.235-1.998	0.488
Tumor stage	Stage I	—	—	—
	Stage II	1.024	0.278-3.768	0.972
	Stage III	1.818	0.397-8.320	0.441
ECOG	(0 or 1)	1.019	0.286-3.631	0.977
Smoking history	(No, Yes)	1.378	0.49-3.879	0.543
Alcohol history	(No, Yes)	0.873	0.276-2.768	0.818
BMI	(<24 or ≥24)	1.189	0.429-3.292	0.739
Pathological type	(Squamous cell carcinoma, adenocarcinoma)	0.678	0.214-2.150	0.678
Tumor location	(left, right)	0.359	0.101-1.280	0.114
Therapeutic evaluation	(SD, PR)	2.407	0.75-7.729	0.14
R0 resection	(Yes, No)	4.302	1.161-15.937	0.029
pCR	(Yes, No)	0.008	1.719-33.901	0.008
MPR	(Yes, No)	0.267	0.091-0.782	0.016
NLR	(High, Low)	0.419	0.118-1.493	0,18
PLR	(High, Low)	5.415	1.519-19.30	0.009
SII	(High, Low)	0.236	0.031-1.802	0.164
PNI	(High, Low)	0.257	0.033-2.985	0.193
Count score	(High, Low)	1.189	0.335-4.216	0.789

**Table 8:** Multivariate analysis of EFS.

Clinical features	Multivariate analysis		
	HR	95%CI	*P*
R0 resection(Yes, No)	0.464	0.120-1.788	0.264
pCR(Yes, No)	0.125	0.019-0.809	0.029
MPR(Yes, No)	0.720	0.181-2.872	0.642
PLR(High, Low)	4.712	1.250-17.756	0.022

#### 3.6.8 Nomogram and calibration curve

**Figure 4:**
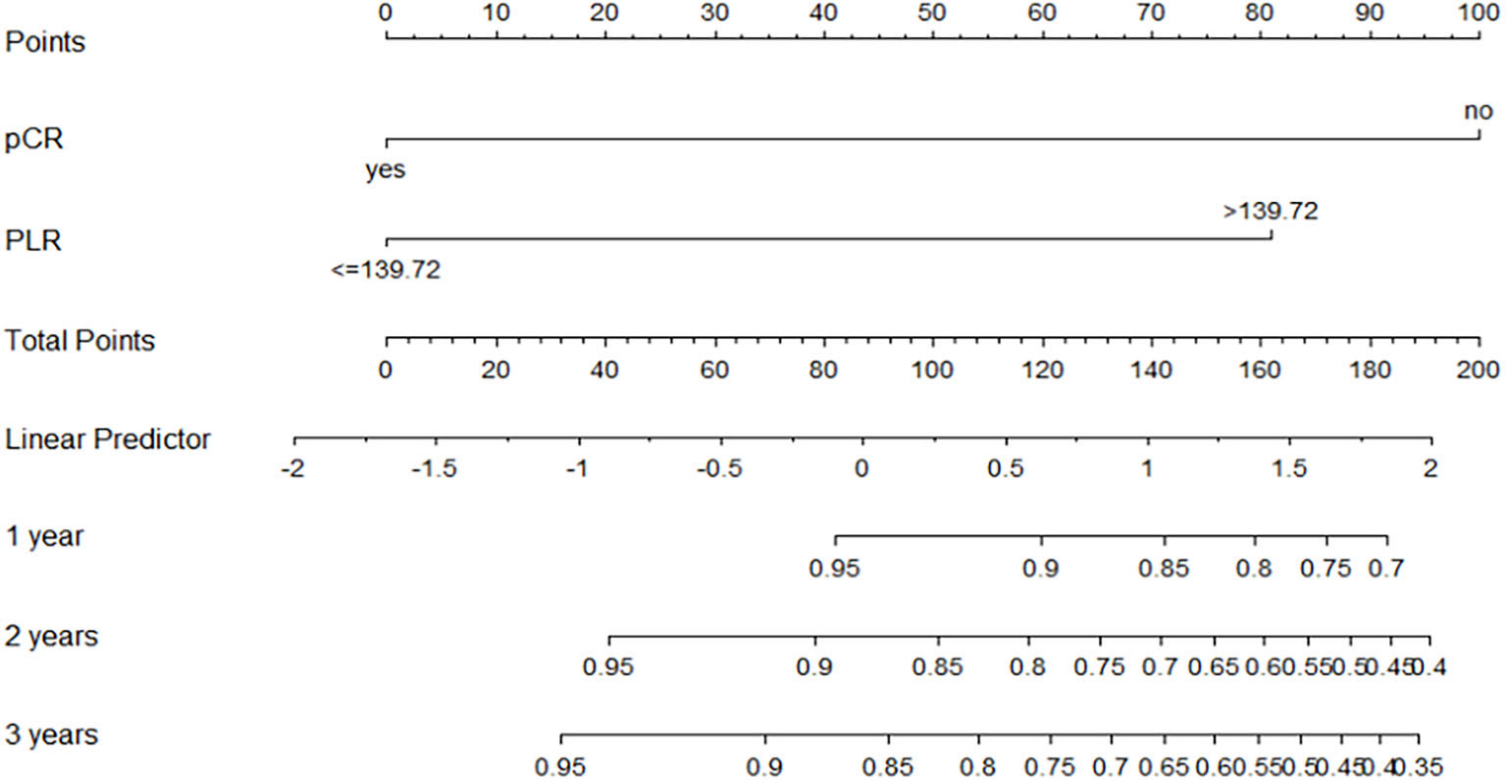
Nomogram for predicting EFS at 1, 2, and 3 years.

**Figure 5:**
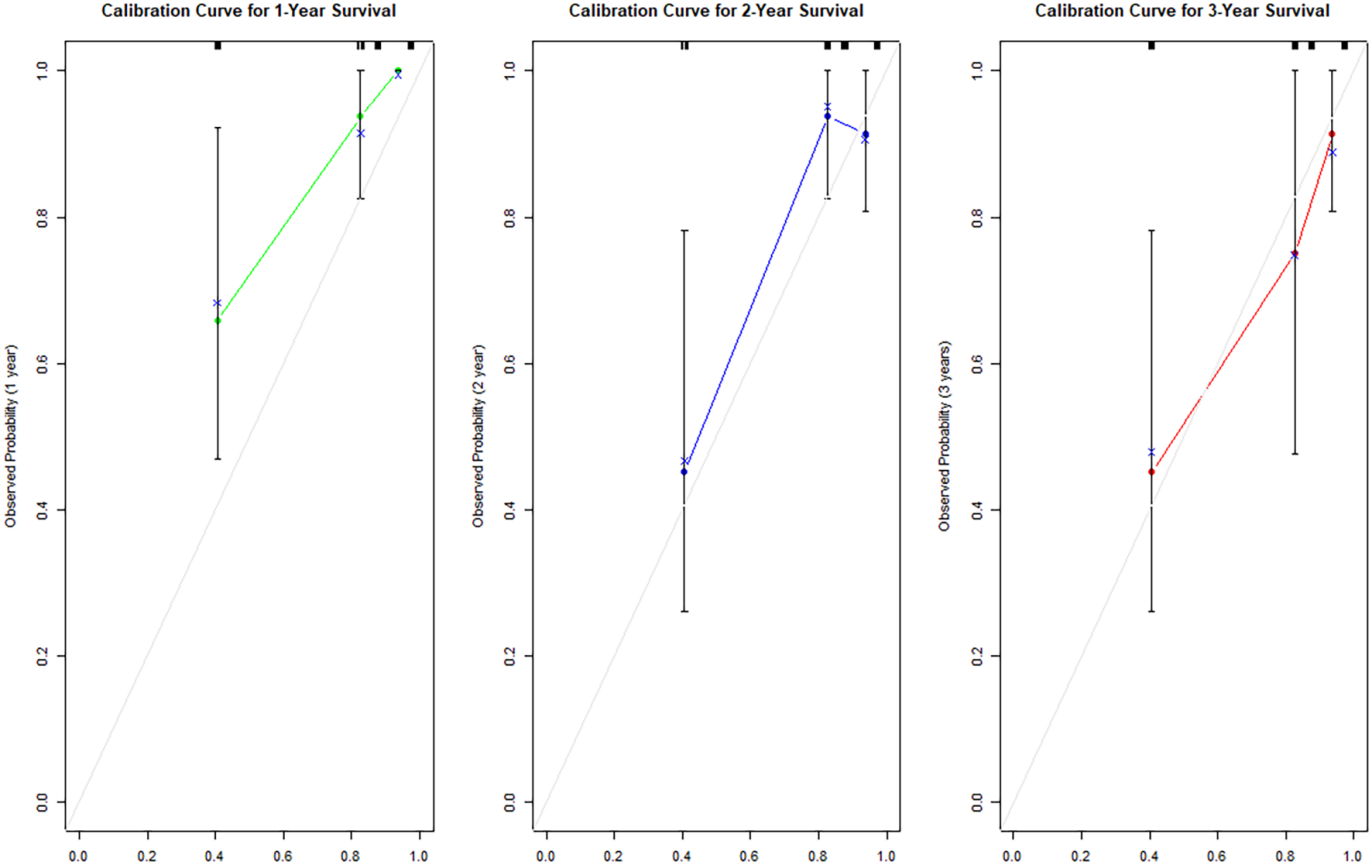
Calibration curves for 1, 2, and 3 years.

Based on the multivariable COX regression model, we further incorporated significant variables from the multivariable analysis into a scaled Nomogram model to predict the prognostic risk of progression of EFS at 1, 2, and 3 years in NSCLC patients (see **Figure 4**). The performance of the predictive model was assessed using the concordance index (C-index), where values ranging from 0.5 to 1 indicate good model performance. A Calibration curve was plotted to illustrate the accuracy of the model, with the closer the prediction curve is to the 45-degree diagonal line, the better the fit. Calibration curve analysis and visualization were performed using R software (version 4.2.3) with the “rms” package. The results indicated that the C-index was 0.803 (95% CI: 0.707–0.897). As shown in **Figure 5**, at a 3-year follow-up, the calibration curve closely approximates the 45-degree diagonal line, whereas the calibration curves for the first and second years exhibited some deviation from the diagonal line compared to the 3-year follow-up.

#### 3.6.9 Time-dependent Receiver Operating Characteristic Curve

**Figure 6:**
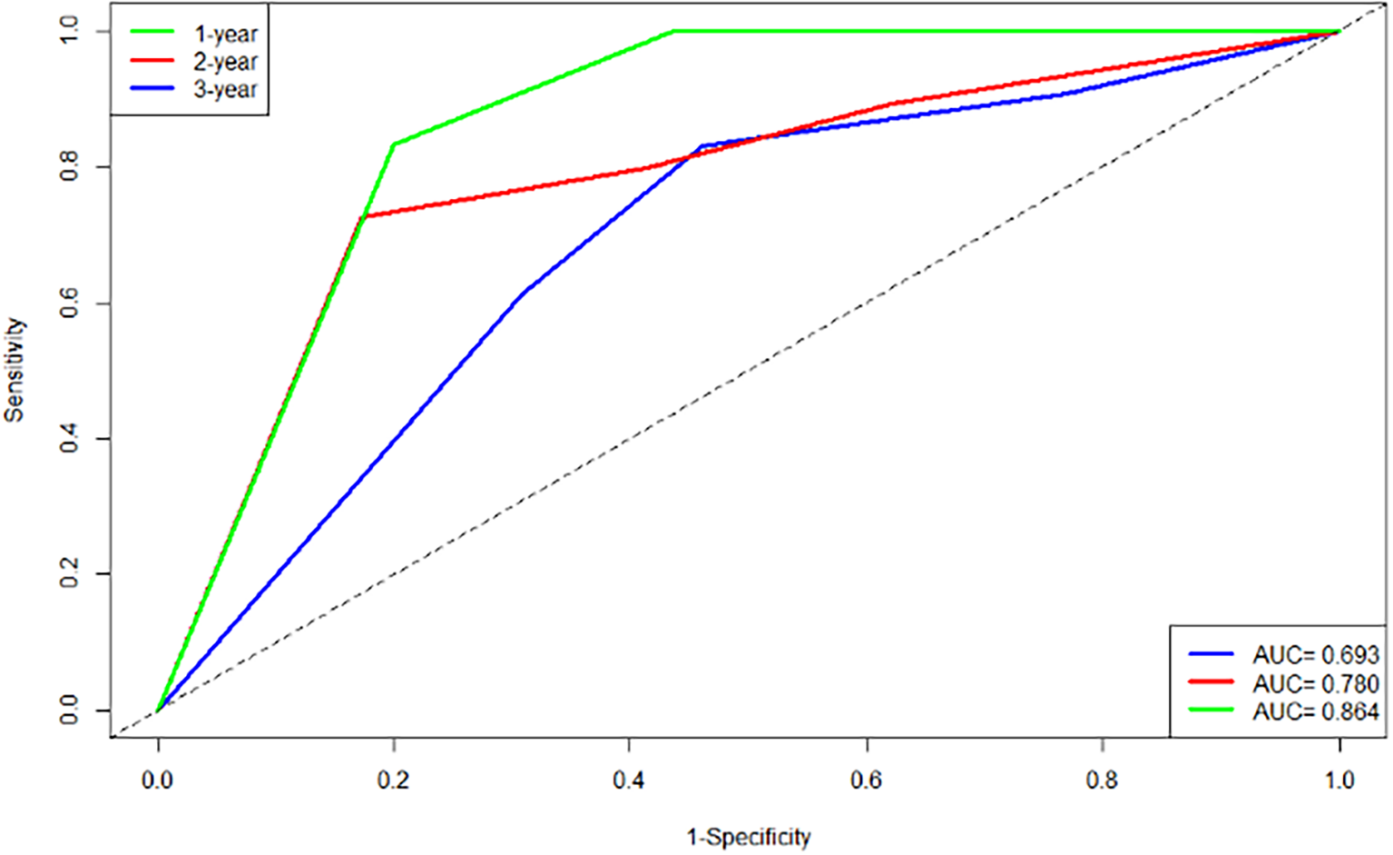
Time-ROC curves at 1, 2, 3 years.

The area under the time-dependent ROC curve for the predictive model at 1, 2, and 3 years were 0.864, 0.780, and 0.693, respectively (**Figure 6**)

### 3.7 Decision Curve Analysis

**Figure 7:**
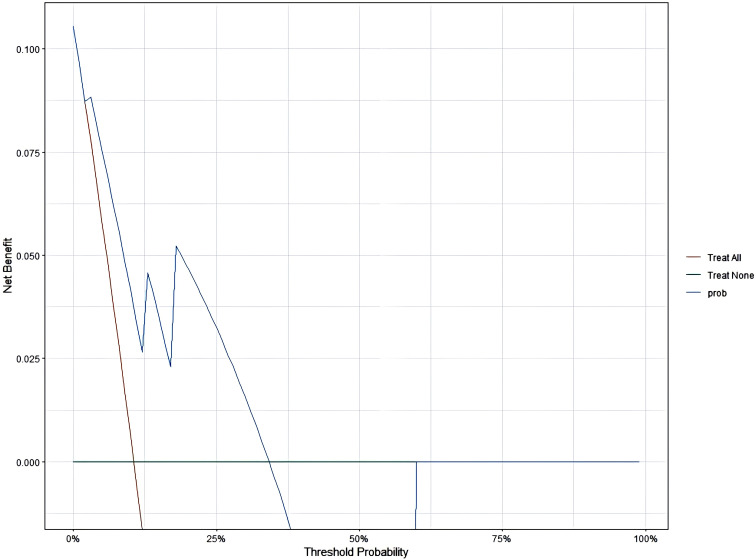
DCA for 1 year.

**Figure 8:**
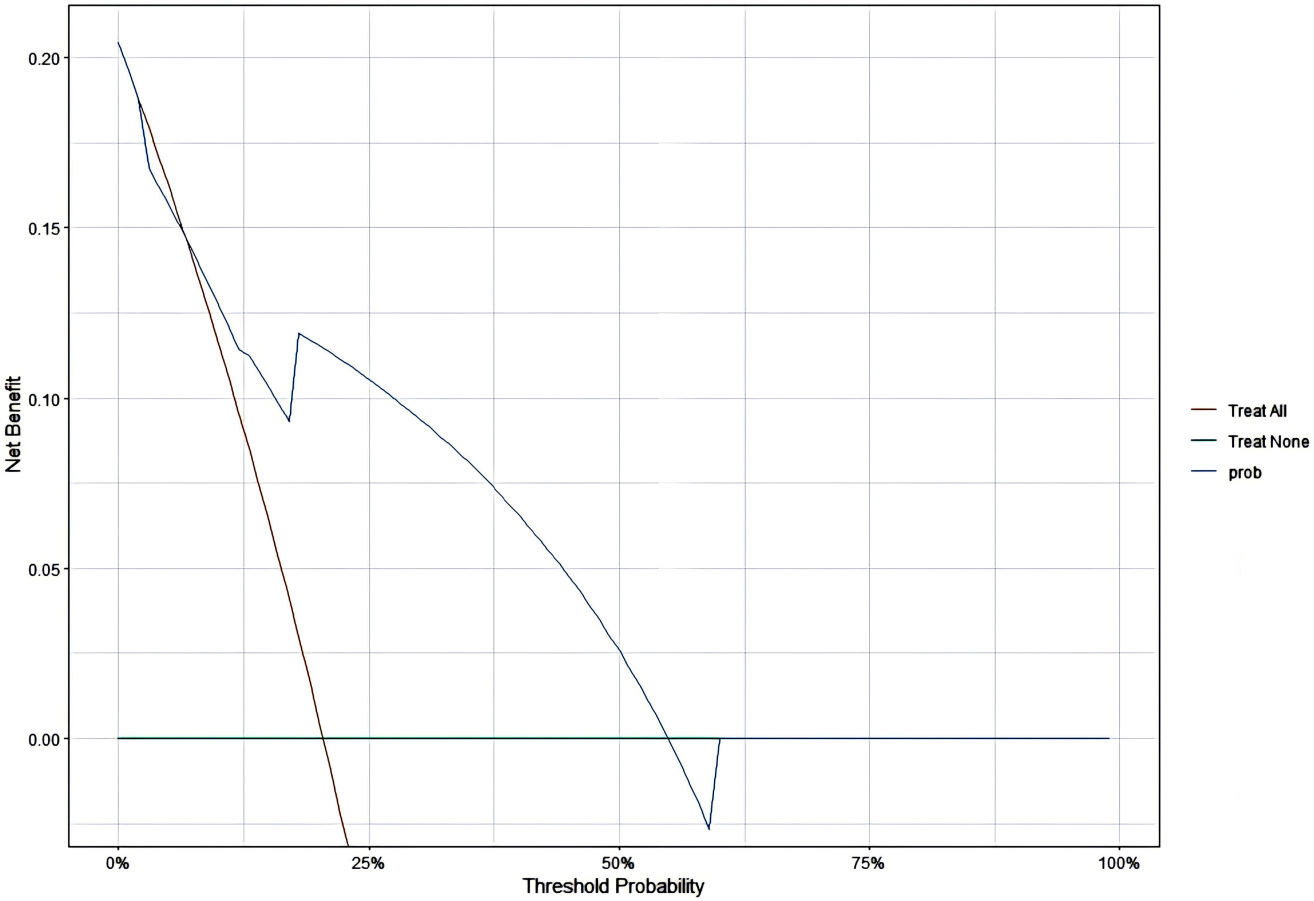
DCA for 2 years.

**Figure 9:**
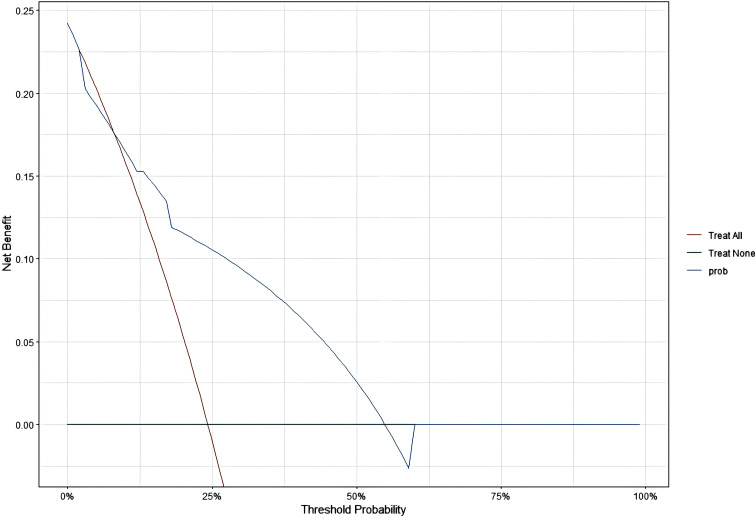
DCA for 3 years.

The model demonstrated that the DCA for 1, 2, and 3 years exhibited net benefits consistently higher than the “all positive” and “all negative” lines within a certain range, indicating the model’s clinical applicability (**Figures 7**–**9**).

## 4 Discussion

Cancer remains a leading cause of mortality worldwide, with a multitude of etiological factors contributing to its development. Lung cancer, particularly NSCLC, has one of the highest incidence rates among malignancies, accounting for approximately two-thirds of all lung cancer cases. Historically, conventional chemoradiotherapy has been the cornerstone of treatment for NSCLC patients. However, with the recent advent of immune checkpoint inhibitors, a novel therapeutic approach has emerged, offering patients extended survival and improved outcomes. This study focuses on patients with stage I-III NSCLC, aiming to evaluate the efficacy and safety of neoadjuvant chemoimmunotherapy in real-world settings. By comparing pathological responses, radiological responses, and EFS between patients receiving neoadjuvant chemotherapy and those receiving neoadjuvant chemoimmunotherapy, we sought to provide insights into the relative advantages of these treatment strategies. In addition, this study further explored the efficacy and influencing factors of neoadjuvant chemotherapy combined with immunotherapy and established a prognostic model to predict the outcomes of these patients.

In the NEOSTAR and NADIM trials, the MPR rates were reported as 50% and 83%, respectively, while the pCR rates were 30% and 63%, respectively (7, 13). Although these clinical trials have robustly demonstrated the efficacy and safety of neoadjuvant chemoimmunotherapy, most of these studies were phase II trials, with the majority of participants being stage IIIA or earlier. In real-world settings, factors such as patients’ physical conditions, comorbidities, and other clinical variables may exclude some individuals from randomized controlled trials (RCTs). Consequently, real-world studies provide a more comprehensive reflection of patients’ actual responses and outcomes during treatment. Compared with traditional clinical trials, real-world cohorts are more diverse, including patients with poorer performance status or other limiting factors. This diversity enhances the clinical applicability of the findings and facilitates a more thorough evaluation of the adaptability and efficacy of treatment regimens across different patient populations. Moreover, existing data reveal considerable variation in the overall efficacy of neoadjuvant chemoimmunotherapy, highlighting an urgent need to identify subgroups of patients who may benefit most from this therapeutic approach. This remains a critical challenge warranting further investigation. In our study, the pCR and MPR rates in the neoadjuvant chemoimmunotherapy group differed from those reported in previous clinical trials. This discrepancy may stem from the heterogeneity of patients’ physical conditions in real-world settings, the broader inclusion criteria employed in our study, and the enrollment of patients with more advanced stages, including stage IIIB and IIIC. Notably, the pCR rate in the neoadjuvant chemoimmunotherapy group was significantly higher than that in the chemotherapy-only group (48.8% vs. 12.5%, *P* < 0.05), underscoring the potential of immunotherapy in neoadjuvant treatment.

While no statistical difference in MPR was observed between the two groups (36% vs. 27.1%, *P* > 0.05), the significant difference in pCR (48.8% vs. 12.5%, *P* < 0.05) highlights the promising role of immunotherapy in enhancing pathological outcomes for patients with stage I-III NSCLC. Although the MPR rate in the chemoimmunotherapy group was higher than that in the chemotherapy-only group, statistical significance might not have been reached due to factors such as sample size, patient heterogeneity, and treatment adherence. Despite the lack of statistical significance in MPR, the significant improvement in pCR indicates the strong therapeutic potential of immunotherapy in this patient population. The inclusion of immunotherapy enhanced patients’ long-term treatment outcomes. Although the initial effects might not appear pronounced, prolonged treatment yielded significant improvements in patients’ long-term prognoses. Therefore, immunotherapy plays a vital role in extending EFS for patients with NSCLC.

Moreover, in the NEOSTAR study, the ORR was 73.9%, and the R0 resection rate reached 95% (7). Our study results are largely consistent with these findings. Although there was no significant difference in R0 resection rates between the two groups in our study (93% vs. 89.6%, *P* > 0.05), this may be attributed to individual variations during treatment and other clinical factors, such as tumor staging and type. These findings underscore the importance of developing personalized treatment plans in clinical practice. The incorporation of immunotherapy not only improved direct therapeutic outcomes but also potentially delayed tumor recurrence and metastasis, leading to longer EFS. This was validated in the Kaplan-Meier survival curve, where the neoadjuvant chemotherapy combined with immunotherapy group significantly prolonged the median EFS (EFS not reached vs. control group 33 months, HR = 0.45), reducing the risk of disease progression, recurrence, or metastasis by 55% (*P* < 0.05). These results further highlight the critical role of immunotherapy in the treatment of stage I-III NSCLC.

Regarding adverse events, the NADIM study was the first multicenter, single-arm, phase II clinical trial to explore the efficacy and safety of neoadjuvant chemoimmunotherapy in stage IIIA (N2) NSCLC patients. In this study, 46 patients were scheduled to receive three cycles of nivolumab combined with platinum-based doublet chemotherapy as neoadjuvant treatment. The results showed that 43 out of 46 patients (93%) experienced TRAEs, and 14 patients (30%) had grade ≥3 adverse events. However, none of these adverse events caused surgical delays or death. The most common grade ≥3 adverse events were elevated lipase levels (3 patients, 7%) and febrile neutropenia (3 patients, 7%) (10). Our study data are consistent with previous clinical trials, showing that the severity of most adverse reactions is manageable and does not significantly impact the treatment process of patients. This indicates that the side effects of immune checkpoint inhibitors in combination with chemotherapy can be effectively controlled through proper management. Therefore, although immunotherapy may increase the occurrence of irAEs, its overall acceptability and controllability make it an important treatment option for patients with stage I-III NSCLC.

Exploring prognostic factors for patients with stage I-III NSCLC undergoing neoadjuvant chemoimmunotherapy holds significant clinical value. On one hand, the goal of neoadjuvant therapy is to improve tumor resectability before surgery, reduce the risk of micrometastases and recurrence, and enhance long-term outcomes. Identifying prognostic factors can facilitate more precise evaluation of treatment efficacy and optimize patient selection. On the other hand, compared to stage IV advanced patients, the evaluation of immunotherapy efficacy in stage I-III NSCLC remains insufficiently studied, particularly in real-world settings where potential biological predictive markers have not been fully established. Previous studies have shown that peripheral blood inflammatory biomarkers can dynamically reflect treatment response and predict prognosis in NSCLC patients undergoing neoadjuvant immunotherapy (12). Additionally, for stage III and IV NSCLC patients, certain inflammatory markers, serum tumor markers, and nutritional indices have been identified as independent prognostic factors (14). However, most of these studies focus on advanced lung cancer patients or those receiving chemotherapy alone. Whether these markers are equally applicable to neoadjuvant chemoimmunotherapy remains unclear. Therefore, this study not only fills this gap but also provides new insights into optimizing treatment strategies for stage I-III NSCLC patients. This study selected NLR, PLR, SII and PNI as predictive markers based on their biological relevance and accessibility. NLR and PLR reflect tumor-associated inflammatory status, which is known to play a crucial role in cancer progression and immune evasion. SII, a composite index based on NLR and PLR, provides a more comprehensive assessment of systemic inflammation levels. PNI reflects patients’ overall nutritional status and immune function. The study aimed to investigate whether clinical characteristics and these inflammatory and nutritional markers (NLR, PLR, SII, and PNI) are independent influencing factors for MPR, pCR, and EFS. The findings revealed that these markers were not effective predictors for MPR. However, pathological subtype was identified as an independent prognostic factor for pCR, with squamous cell carcinoma patients being more likely to achieve pCR. This observation aligns with previous studies. For instance, the KEYNOTE-021 trial evaluated the efficacy of pembrolizumab (Keytruda) combined with chemotherapy in NSCLC patients and reported a significantly higher pCR rate in patients with squamous cell carcinoma compared to those with adenocarcinoma. This may be attributed to the higher immunogenicity of squamous cell carcinoma and its greater sensitivity to immune checkpoint inhibitors (12). Numerous factors may be closely associated with tumor response and prognosis. Clinical characteristics, treatment regimens (e.g., specific chemotherapy and immunotherapy drugs), and other molecular biomarkers (e.g., PD-L1 expression, genetic mutations) could all have a significant impact on MPR and pCR outcomes. These factors may obscure the independent prognostic value of NLR, PLR, SII, and PNI. Furthermore, the combined use of chemotherapy and immunotherapy may involve complex interactions, limiting the ability of these hematological markers to independently predict efficacy.

Nevertheless, for EFS in patients undergoing neoadjuvant chemoimmunotherapy, statistical analysis demonstrated that pCR and PLR were independent prognostic factors for EFS.

Additionally, this study established a nomogram model to predict the EFS of stage I-III NSCLC patients undergoing neoadjuvant chemoimmunotherapy based on the results of the Cox regression analysis.1.Calibration Curve Analysis shows the calibration curve analysis demonstrated that the model exhibited high accuracy in predicting 3-year EFS, effectively reflecting the long-term prognosis of patients. However, at 1-year and 2-year follow-ups, the calibration curves showed some deviation from the 45-degree diagonal line. This deviation could be attributed to insufficient sample size and the low incidence of adverse events during the early follow-up period. For instance, among stage I-III NSCLC patients receiving immunotherapy during the neoadjuvant phase, EFS was prolonged, and the number of patients experiencing disease progression in the short term was relatively low. As the duration of treatment extended (e.g., 3 years), the number of progression events gradually increased, improving the predictive capability of the model and aligning the calibration curve closer to the diagonal line.2.ROC Curve Analysis revealed that the model exhibited strong predictive performance for 1-year, 2-year, and 3-year EFS. 3.DCA curve analysis indicated that the model provided a net benefit compared to conventional treatment strategies and no-treatment approaches during the 1-year, 2-year, and 3-year follow-up periods. Notably, the model offered significant clinical benefits in predicting EFS during the first and second years. During these early years, the number of patients experiencing disease progression was relatively low, allowing the DCA to effectively identify high-risk patients and provide greater clinical benefits.

However, by the third year, the number of patients with disease progression increased. The model’s predictive accuracy diminished, potentially due to the complexity of prognostic changes, such as immune tolerance and alterations in the tumor microenvironment, which may reduce the effectiveness of clinical decision-making.

Additionally, during the course of immunotherapy, treatment regimens may be adjusted based on patient responses. In the first and second years, patients exhibited relatively stable responses to neoadjuvant chemoimmunotherapy, enabling the model to better predict adverse events. However, as time progressed (especially into the third year), treatment strategies often underwent modifications, such as changes in therapy duration or the addition of other medications. These factors might not have been fully captured by the model, potentially impacting its predictive performance and clinical applicability in the third year.

There are some advantages of this study: (1) Single-Center Design: This study’s single-center design ensures consistency in patient treatment and follow-up, providing a reliable framework for reproducible data collection. (2) Real-World Data: The study offers valuable real-world evidence on the efficacy and adverse events of neoadjuvant chemoimmunotherapy for stage I-III NSCLC patients, providing clinically relevant insights. (3) Cost-Effective Predictive Indicators: The selected predictive biomarkers are inexpensive and easy to assess. While these indicators have primarily been used to predict outcomes in advanced-stage patients in previous studies, this research fills a gap by evaluating their utility in stage I-III NSCLC patients undergoing neoadjuvant chemoimmunotherapy. (4) Predictive Model Development: Based on Cox regression analysis, this study developed a predictive model for EFS in stage I-III NSCLC patients treated with neoadjuvant chemoimmunotherapy. The model demonstrated high accuracy and offers potential clinical value.

There alos have some limitations of this study. (1) Retrospective Nature: As a retrospective study, it is prone to selection bias and potential issues with data completeness. (2) Limited Sample Size: The relatively small sample size, particularly for subgroup analyses, may limit statistical power. (3) Short Follow-Up Duration: The study’s follow-up period was relatively short, preventing the assessment of long-term outcomes, such as 5-year survival rates. (4) Incomplete PD-L1 Data: Data on PD-L1 expression, a well-established clinical predictor, were incomplete, making it challenging to validate this biomarker’s role in a real-world setting. (5) Suboptimal Predictive Indicators: The predictive biomarkers selected in this study demonstrated limited efficacy. Future research should incorporate additional biomarkers to identify high-performance and clinically applicable predictors for the effectiveness of neoadjuvant chemoimmunotherapy in stage I-III NSCLC patients.(6)The study shows an imbalance in the proportion of squamous cell carcinoma and adenocarcinoma. In the future, efforts should be made to increase the data volume.

In summary, this study provides real-world insights into comprehensive treatment strategies for stage I-III NSCLC, particularly the efficacy and safety of neoadjuvant chemoimmunotherapy. The identification of inflammation- and nutrition-related biomarkers as potential prognostic factors offers further guidance for personalized treatment. Future research should aim to expand the sample size and extend the follow-up duration to validate these findings and optimize therapeutic strategies.

## 5 Conclusion

1. In the real-world setting, for stage I-III NSCLC patients, compared to neoadjuvant chemotherapy alone, neoadjuvant chemotherapy combined with immunotherapy offers superior pathological complete response rates, radiological objective response rates, and EFS. Furthermore, the incidence of adverse reactions during the neoadjuvant treatment phase was lower, with manageable severity.
2. Pathological type is an independent prognostic factor for pCR (squamous cell carcinoma patients are more likely to achieve pCR).
3. pCR and PLR are independent prognostic factors for EFS.
4. Based on COX analysis, a prognostic model for EFS in stage I-III NSCLC patients undergoing neoadjuvant chemotherapy combined with immunotherapy was established. This model demonstrated good accuracy and has potential clinical application value.

## Data Availability

The original contributions presented in the study are included in the article/supplementary files. Further inquiries can be directed to the corresponding author.
